# Intramammary lipopolysaccharide infusion alters the fatty acid composition of blood lipid fractions but not milk in dairy cows

**DOI:** 10.1186/s40104-025-01272-z

**Published:** 2025-10-23

**Authors:** Chrissy Lalonde, Jana Kraft, Ratan K. Choudhary, Erin M. Shangraw, Thomas B. McFadden, Feng-Qi Zhao

**Affiliations:** 1 Department of Animal and Veterinary Sciences, University of Vermont, Burlington, VT 05405 USA; 2 Department of Medicine, Division of Endocrinology, Metabolism, and Diabetes, University of Vermont, Colchester, VT 05446 USA; 3 Division of Animal Science, University of Missouri, Columbia, MO 65211 USA

**Keywords:** Desaturation index, Mastitis, Prostaglandin, Systemic effect, Triacylglycerides

## Abstract

**Background:**

Mastitis is known to alter milk lipid yield, but its effects on lipid composition in blood and milk remain less understood. This study investigated changes in fatty acid (FA) composition in blood lipid fractions and milk of dairy cows following an intramammary lipopolysaccharide (LPS) challenge and explored potential links associated with these changes. We hypothesized that intramammary LPS infusion would alter the FA composition of blood lipid fractions, and that milk FA composition would reflect these changes. Furthermore, we hypothesized that prostaglandin E_2_ (PGE_2_) would be associated with changes in both blood and milk FA composition, functioning as a potential mediator of these changes.

**Results:**

Ten lactating cows were split into two groups. The treatment group received intramammary infusions of 50 μg *Escherichia coli* LPS in both quarters of one udder half to induce clinical mastitis, and saline infusions in the quarters of the opposite udder half; the control group received saline infusions in one udder half only. Blood and foremilk were collected from individual cows or glands at −1, 3, 6, 12, and 24 h post-infusion. Blood lipids were fractionated into cholesterol esters, free fatty acids (FFA), phospholipids (PL), and triacylglycerides (TAG). The FA composition was analyzed via gas-liquid chromatography. Total plasma TAG, FFA, and PGE_2_ concentrations were measured by colorimetric assay or ELISA. Statistical significance was determined using mixed models with Tukey’s test. Lipopolysaccharide infusion did not affect total plasma TAG and FFA concentrations but increased plasma PGE_2_ concentrations and Δ9 desaturation indices in plasma TAG. A distinct shift in FA composition in plasma phospholipids and TAG was observed between the treatment and control groups at 6 and 12 h post-infusion. Specifically, LPS increased the proportion of n-6 polyunsaturated FA (18:2, 18:3, 20:3, 20:4, 20:5) and FA with less than 16 carbons while decreasing the saturated FA (18:0 and 20:0) in plasma TAG at 6 and 12 h. However, the milk FA composition remained unchanged.

**Conclusion:**

Our findings indicate that transient intramammary LPS challenge influences systemic lipid metabolism without altering the milk FA composition, suggesting that mammary inflammatory responses affect blood lipids independently of milk lipid secretion.

## Background

Mastitis, the inflammation of the mammary gland, is the most economically significant disease affecting dairy cattle [1, 2]. The associated costs primarily stem from reduced milk yield and quality [3–5]. Bacterial mastitis is caused by Gram-positive species, such as *Streptococcus* spp*.* and S*taphylococcus* spp*.,* or by Gram-negative species, including *Escherichia coli, Klebsiella* spp*.*, and *Pseudomonas* spp*.* [2, 6]. The hallmark of Gram-negative bacteria is their cell-wall structure, which includes an outer membrane rife with lipopolysaccharides (LPS) [7]. Lipopolysaccharides are the main inflammatory agents of Gram-negative mastitis and are widely used to induce experimental mastitis in mice and cows [8, 9].

Milk lipids are composed of about 98% of triacylglycerides (TAG), made up of fatty acids (FA) derived from two sources: de novo synthesis in the mammary gland (short- to medium-chain FA, SMCFA) and direct uptake from blood (medium- to long-chain FA) [10]. Long-chain FA (LCFA) primarily originate from the hydrolysis of blood TAG by lipoprotein lipase but can also come from circulating free FA (FFA) [11]. Thus, milk LCFA are thought to be influenced by blood lipid profiles, which derive from the diet, rumen microbial metabolism, and lipid metabolism in hepatocytes and adipocytes. Beyond their role in milk lipid synthesis, blood lipids are also essential for overall homeostasis, such as serving as energy sources, precursors for potent inflammatory mediators (e.g., prostaglandins and leukotrienes), and regulators of metabolic and inflammatory processes [12, 13].

Mastitis alters milk composition, including milk lipid content in moderate to severe mastitis cases [5, 14]. We have previously demonstrated that an intramammary LPS infusion in one udder half reduced the total fat content in the milk of all four quarters within 12 h post-infusion [5], suggesting that changes in milk fat content resulted from local mammary-specific effects as well as systemic animal contributions. Clinical mastitis is associated with reduced milk fat yield [15, 16] and, generally, an increase in FFA content in milk [17]. However, reports on mastitis-induced changes in milk fat percentage are inconsistent, showing increases [18], decreases [19], or no effects [20] likely due to variations in mastitis severity and etiology [21]. By comparison, the effects of mastitis on FA composition in milk and blood lipid fractions remain poorly characterized. In blood, FA are primarily found in four forms: FFA, cholesterol esters (CE), glycerolipids (monoacylglycerols, diacylglycerols, and TAG), and phospholipids (PL). While mastitis has been shown to alter the composition of the FFA fraction of blood lipids, the impact on other fractions remains largely underexplored [22]. Studies on total blood lipids suggest that mastitis increases the proportion of oleic acid (18:1 9*c*) and linoleic acid (18:2 9*c*,12*c*) in plasma and modulates bioactive oxylipins [23]. Oxylipins, highly oxidized lipids derived from polyunsaturated fatty acids (PUFA), act as chemical messengers and play roles in a wide variety of biological functions [24]. One major function of oxylipins is the regulation of inflammation [25], and as such, they could be powerful drivers of mastitis severity, such as fever, nociception, edema, and vasodilation. Given their role in inflammatory processes, oxylipins likely contribute to the systemic effects of mastitis [12, 26]. However, the extent to which mastitis alters the FA composition in both blood lipid fractions and milk and the physiological significance of these changes remain poorly understood.

This study aimed to determine the effects of an intramammary LPS challenge on the FA composition of blood lipid fractions and milk in lactating dairy cows. We hypothesized that mastitis would alter plasma FA composition, and these changes would be reflected in the milk FA composition. In addition, we hypothesized that the plasma prostaglandin E_2_ (PGE_2_), a major oxylipin, would correlate with the changes in both milk and plasma FA composition.

## Materials and methods

### Animals and experimental design

All procedures involving animals in this study were approved by the Institutional Animal Care and Use Committees of the University of Vermont (protocol No. 17–028) and the University of Missouri (protocol No. 9283). The animal experimental design and management were previously described in detail [5]. Briefly, the study included eight Holstein-Friesian and two Holstein-Jersey multiparous cows (parity 2–5). All cows had a somatic cell count (SCC) below 174,000 cells/mL and were free of major mastitis pathogens, as confirmed by bacterial culture of foremilk strippings collected 14 and 8 d prior to the experiment. Cows were milked every 12 h, had free access to water, and were fed a total-mixed ration formulated to meet lactation requirements ad libitum.

Cows were paired by breed and milk yield, with one cow from each pair randomly assigned to either the LPS treatment or control group. Each group included two experimental conditions. In the LPS treatment group, both quarters of one udder half were injected with 50 µg of LPS from* E. coli* O55:B5 (Sigma-Aldrich, St. Louis, MO, USA) in 10 mL of sterile saline (TL) while the remaining two quarters received 10 mL of 0.9% sterile saline (TS). In the control group, one udder half was injected with 10 mL sterile saline (CS) and the other half remained untreated (CU). The time of infusion was designated as time 0 h.

### Blood sampling

Blood samples were taken from the coccygeal vein at −1 (designated as time zero), 3, 6, 12, and 24 h relative to the intramammary challenge. Samples were drawn into Vacutainer^®^ tubes with K3 ethylenediaminetetraacetic acid (EDTA) for plasma collection. Blood for plasma separation was centrifuged within 5 min of collection at 2,200 × *g* for 15 min at 4 °C. The resulting supernatant was aliquoted and stored at −80 °C until further analysis.

### Foremilk collection

At −1, 3, 6, 12, and 24 h post-intramammary LPS challenge, teats were dipped in iodine and thoroughly dried with a paper towel before sample collection. Subsequently, 25 mL of foremilk was collected through the teat canal and sent to Dairy Herd Information (Mid-South Dairy Records, Springfield, MO, USA) for component analysis. An additional 85 mL of foremilk was collected, half of which was used for FA analysis and processed as described below.

### Plasma free fatty acid and triacylglyceride concentration analyses

Plasma FFA concentration was measured with the Fujifilm HR series NEFA-HR (2) kit (#999–34691, Wako Chemicals, Chuo-Ku, Japan). Briefly, 1 mL of non-esterified FA (NEFA) standard (#270–77000, Wako Chemicals) was mixed with 1 mL of distilled water, and a standard curve was generated by linear regression from a series of dilutions of the standard solution. In a 96-well plate, 80 µL of color reagent A and 8 µL of either plasma or the NEFA standards for the standard curve and for the inter-plate standard were mixed and incubated for 10 min at 37 °C. Then, 160 µL of color reagent B was added to each well, and the plate was gently mixed at 37 °C for another 10 min. Absorbance was measured at 550 nm using a Synergy HT plate reader (BioTek Instruments, Winooski, VT, USA). All samples and standards were analyzed in triplicate. A blank value was subtracted from all sample and standard absorbance values. The assay’s effective measurement range was 0.01 to 4.00 mEq/L with an accuracy of ± 15% and difference of 6.04% per sample between replicates.

Plasma TAG concentrations were measured using the Triglyceride Colorimetric Assay Kit (#10010303, Cayman Chemicals, Ann Arbor, MI, USA) according to the manufacturer’s protocol using undiluted plasma. One in two serial dilutions of TAG standards were made from 200 mg/dL to 3.125 mg/dL, and a standard at 0 mg/dL was also made using only the kit’s standard dilutant. Absorbance was measured at 540 nm on a Synergy HT plate reader. The 0 mg/dL absorbance value was subtracted from the values of all samples and standards. All samples and standards were analyzed in triplicate, and the concentrations were determined by the standard curve generated through linear regression. The lower limit of detection of the assay was 0.5 mg/dL. The assay’s limit of detection was 0.5 mg/dL with a difference of 3.17% per sample between replicates.

### Plasma and milk FA analysis

Plasma lipids were extracted using methanol-chloroform and separated into four fractions (TAG, FFA, PL, and CE) by solid phase extraction, following the protocol described by Unger et al. [27]. Cream was isolated from milk by centrifugation at 17,800 × *g* for 30 min at 8 °C. To extract total lipids, *n*-hexane/isopropanol solution (3:2 v/v) was added to the cream and the solution was vortexed for 10 min. A 6.7% (w/v) sodium sulfate solution was added, and the mixture was vortexed for an additional 2 min before centrifugation at 1,500 × *g* for 5 min. The upper layer was filtered through sodium sulfate and dried under nitrogen gas. Lipids were reconstituted in *n*-hexane. Methylation of the FA from all plasma fractions and milk was performed according to the methods described by Bainbridge et al. [28]. Methylated FA were analyzed by gas-liquid chromatography on a GC-2010 gas chromatograph (Shimadzu, Kyoto, Japan) and identified by a flame ionization detector as described by Unger et al. [29].

### Data interpretation

Fatty acid identification and integration were performed using GCsolution software version 2.30.00, following the methods described previously [29]. Briefly, the retention time of individual FA was compared to known standards for identification. The reference standards included Nu-Check Prep’s (Elysian, MN, USA) conjugated linoleic acids mixture and standards #463 and #674, Supelco’s (Bellefonte, PA, USA) PUFA mixture 3, Larodan Fine Chemicals’ (Malmö, Sweden) branched-chain FA mixture, and an in-house milk standard. The area under the curve of each peak was calculated, and the relative proportion of each FA was determined as the ratio of its area under the curve to the total area under the curve of all identified peaks. Fatty acids representing less than 0.01% of total FA were excluded from analysis.

### Plasma PGE_2_ analysis

Plasma PGE_2_ concentrations were measured by competitive ELISA using the Prostaglandin E_2_ ELISA Kit Monoclonal (#514010, Cayman Chemicals) on a Synergy HT plate reader according to the manufacturer’s instructions. Plasma concentrations were calculated by comparing the binding percentage of each sample to the log concentrations of a standard curve obtained by serial dilutions of the provided PGE_2_ ELISA standard. The assay’s range of detection was 1.0 to 1,000 pg/mL with an intra-assay variation of 6.6% and an inter-assay variation of 15.5%.

### Statistical analysis

Statistical analyses were conducted using JMP Pro 16 (SAS Institute, Cary, NC, USA). A Mixed Model approach was used with [cow] as a random effect and [Treatment], [Time], and [Treatment × Time] as fixed effects. If the [Treatment × Time] interaction was non-significant, it was removed from the model. Multiple comparisons were performed using Tukey’s Honestly Significant Difference tests. Statistical significance was declared at *P* < 0.05, while trends were noted as 0.05 < *P* < 0.1. Pearson’s correlation coefficients were calculated using JMP Pro 16.

Eigenvalues for principal components analysis (PCA) were determined based on parallel analysis of standardized data (mean = 0, standard deviation = 1) using 1000 Monte Carlo simulations [30]. The first two principal components were used for data visualization, but up to 11 components were calculated and visually verified for clustering patterns. All graphs were generated using GraphPad Prism 9.5 and 10.1 (Boston, MA, USA).

## Results

### Brief summary of milk performance

Following the intramammary LPS infusion, the rectal temperature of the LPS-treated animals increased rapidly during the first 6 h. Milk yield and fat yield gradually decreased over 24 h in both LPS-treated and untreated quarters in the treatment group, whereas the milk fat percentage was reduced during first 6 h. Elevated SCC was only observed in the milk of LPS-challenged gland. Detailed observations of these changes, along with other milk performance data, were previously reported [5].

### Plasma TAG and FFA concentrations

**Fig. 1:**
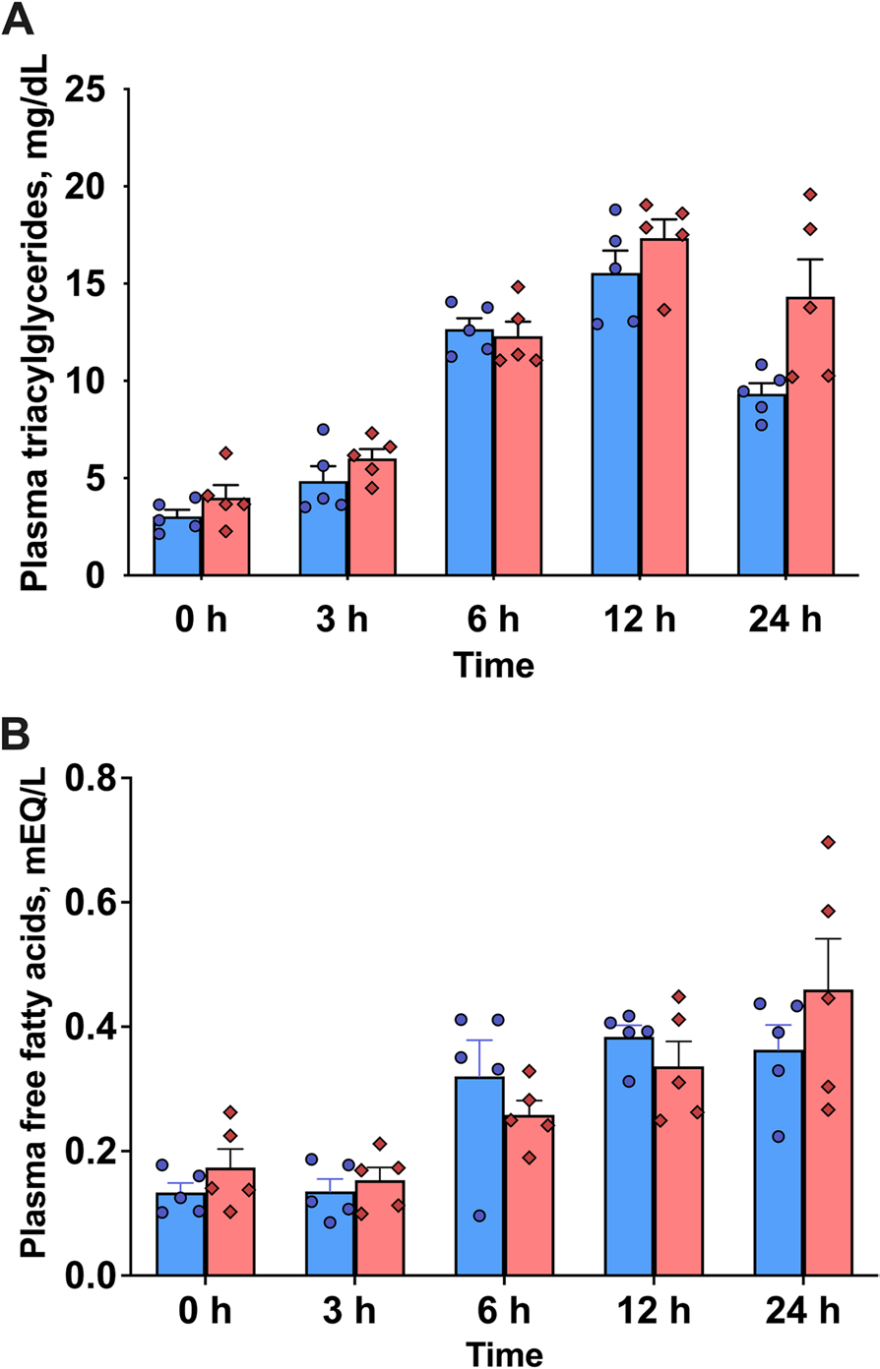
Plasma concentrations of total triacylglycerols (**A**) and total free fatty acids (**B**) in dairy cows following an intramammary infusion of either saline (blue, *n* = 5) or lipopolysaccharide (red, *n* = 5) at 0, 3, 6, 12, and 24 h. There was no treatment effect but there was a significant time effect, and there was no treatment × time interaction in both observations. Error bars represent means ± SEM

No treatment effect was observed for plasma total TAG concentrations in cows intramammarily injected with LPS. However, a strong time effect (*P* < 0.001) was detected (Fig. 1A). No time × treatment interaction was observed (*P* = 0.20). Similarly, plasma total FFA concentrations showed no treatment effect (*P* = 0.43) and no time × treatment interaction (*P* = 0.42). However, a strong time effect (*P* < 0.001) was observed (Fig. 1B).

### Fatty acid composition of plasma TAG

**Fig. 2:**
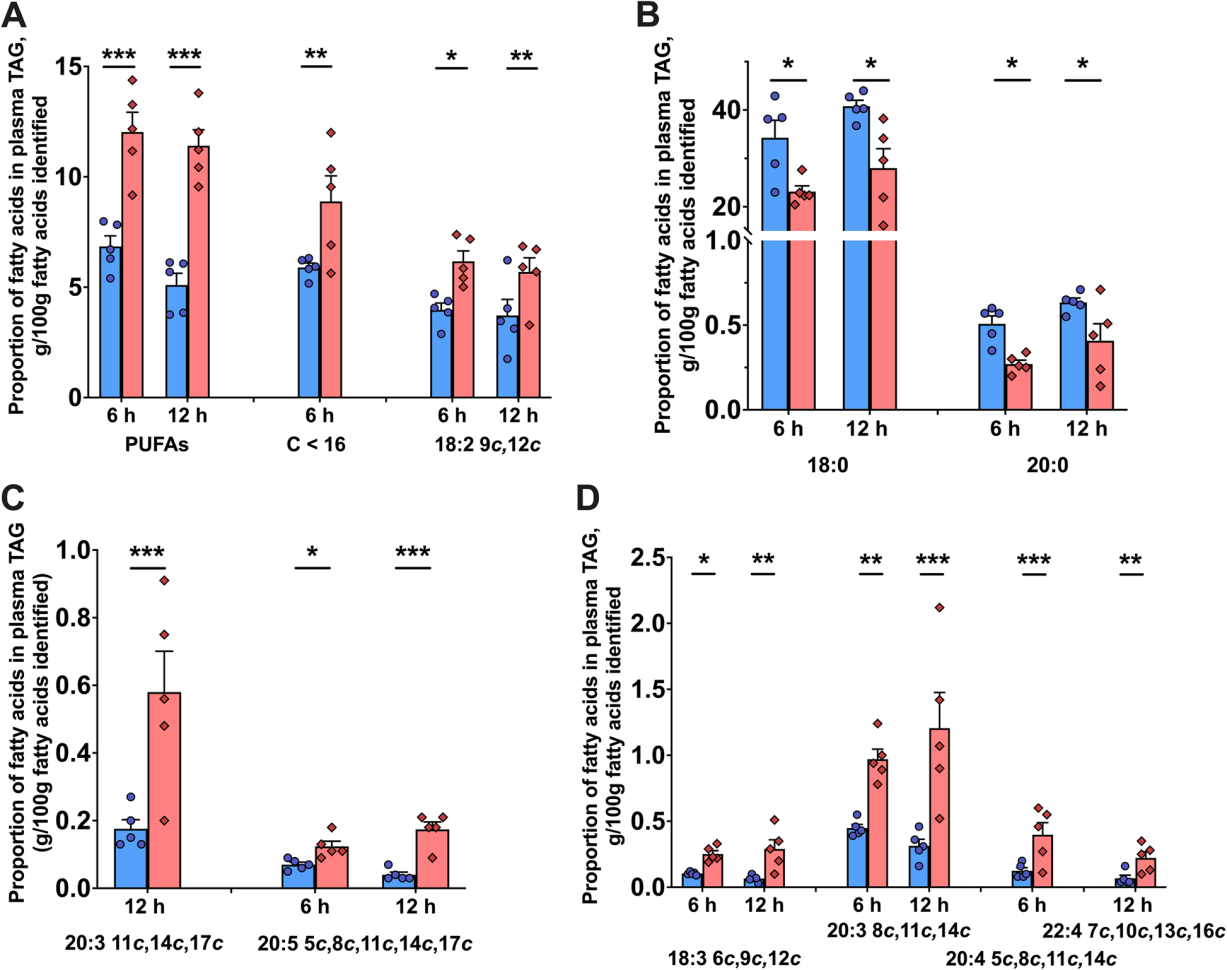
Changes in proportions of individual fatty acids and fatty acid classes in plasma triacylglycerols of dairy cows following an intramammary infusion of either saline (blue, *n* = 5) or lipopolysaccharide (red, *n* = 5) at 6 h and 12 h. **A** Overall fatty acid classes and linoleic acid. **B** Saturated fatty acids. **C** n-3 Fatty acids. **D** n-6 Fatty acids. Error bars represent means ± SEM. ^*^P < 0.05, ^**^P < 0.01, ^***^P < 0.001

Lipopolysaccharide infusion influenced the proportions of PUFA (*P* = 0.004), SMCFA (< 16 carbons, *P* = 0.041) and LCFA (> 16 carbons, *P* = 0.027) in plasma TAG (Table 1). Additionally, a time effect was observed in the proportions of all FA classes in TAG (all *P* ≤ 0.030). Time × treatment interactions were detected for SFA (*P* = 0.006), PUFA (*P* < 0.001), and SMCFA (*P* = 0.018). The proportion of PUFA in plasma TAG increased 1.76-fold at 6 h (*P* < 0.001) and 2.25-fold at 12 h (*P* < 0.001) in LPS-treated cows compared to controls (Table 1 and Fig. 2A). Proportions of SMCFA increased 1.51-fold at 6 h (*P* = 0.006, Table 1). Additionally, LPS-treated cows had a 1.48-fold decrease in 18:0 at 6 h (*P* = 0.038) and a 1.46-fold decrease at 12 h (*P* = 0.011), as well as a 1.89-fold decrease in 20:0 at 6 h (*P* = 0.022) and a 1.54-fold decrease at 12 h (*P* = 0.034, Fig. 2B). Increases were observed for several n-3 FA, including a 3.22-fold increase in 20:3 at 12 h (*P* < 0.001), a 1.71-fold increase in 20:5 at 6 h (*P* = 0.019), and a 4.25-fold increase at 12 h (*P* < 0.001; Fig. 2C). LPS treatment also led to increases in multiple n-6 FA, including 18:2 (1.55-fold increase at 6 h, *P* = 0.017; 1.97-fold increase at 12 h, *P* = 0.001; Fig. 2A), and 18:3 (2.60-fold increase at 6 h, *P* = 0.007; 4.14-fold increase at 12 h, *P* < 0.001; Fig. 2D), 20:3 (2.16-fold increase at 6 h, *P* = 0.028; 3.90-fold increase at 12 h, *P* < 0.001), 20:4 (3.33-fold increase at 6 h, *P* < 0.001), and 22:4 (3.14-fold increase at 12 h, *P* = 0.003; Fig. 2D).

**Table 1:** Proportions [g/100 g fatty acids (FA) identified] of FA within different FA classes across plasma lipid fractions and milk

Lipid fraction and FA type		Time					Pooled	*P*-value		
		0 h	3 h	6 h	12 h	24 h	SEM	Treatment	Time	TRT × Time
Plasma triacylglycerols										
Saturated (SFA)	CTL	70.17	67.11	67.60	73.22	74.59	1.79	0.137	< 0.001	0.006
	LPS	73.59	70.03	59.91	63.84	74.49				
Monounsaturated (MUFA)	CTL	22.66	25.39	25.55	21.68	20.60	1.25	0.877	0.012	0.370
	LPS	20.99	22.58	28.06	24.76	20.41				
Polyunsaturated (PUFA)	CTL	7.17	7.50	6.85	5.10	4.81	0.76	0.004	< 0.001	< 0.001
	LPS	5.42	7.38	12.03^***^	11.40^***^	5.10				
< 16 carbons (SMCFA)	CTL	6.37	6.89	5.89	5.72	5.78	0.36	0.041	0.030	0.018
	LPS	6.63	7.24	8.88^**^	7.00	6.44				
> 16 carbons (LCFA)	CTL	66.70	64.22	65.01	67.20	67.16	0.89	0.027	0.005	0.052
	LPS	66.29	63.37	59.28	62.47	63.76				
Plasma phospholipids										
SFA	CTL	47.43	48.49	49.80	50.21	49.22	0.58	0.310	0.019	0.441
	LPS	47.56	47.52	48.87	48.09	48.17				
MUFA	CTL	12.98	13.54	13.18	13.52	13.97	0.34	0.701	0.004	0.044
	LPS	13.58	13.85	14.61	15.11	14.24				
PUFA	CTL	39.58	37.97	37.02	36.27	36.81	0.64	0.228	< 0.001	0.553
	LPS	38.85	38.63	36.52	36.80	37.59				
SMCFA	CTL	2.34	2.67	3.21	2.99	2.29	0.14	0.066	< 0.001	0.894
	LPS	2.74	2.95	3.20	2.73	2.42				
LCFA	CTL	80.85	79.69	79.10	79.44	79.66	0.28	0.023	< 0.001	0.22
	LPS	79.74	79.14	77.81	78.78	78.65				
Plasma cholesterol esters										
SFA	CTL	9.52	8.86	9.53	9.75	9.48	0.24	0.179	< 0.001	0.002
	LPS	10.06	9.91	10.16	10.09	10.67				
MUFA	CTL	5.27	6.74	5.28	5.14	5.10	0.27	0.624	0.007	0.228
	LPS	5.13	5.64	5.20	5.19	5.30				
PUFA	CTL	85.21	84.41	85.19	85.09	85.42	0.40	0.548	0.359	0.197
	LPS	84.81	84.45	84.61	84.72	84.03				
SMCFA	CTL	3.37	3.02	3.17	3.48	3.30	0.09	0.071	0.010	0.013
	LPS	3.56	3.55	3.68	3.57	3.79				
LCFA	CTL	90.59	91.29	90.76	90.61	90.92	0.18	0.142	< 0.001	0.003
	LPS	90.32	90.39	90.08	90.25	89.94				
Foremilk										
SFA	CS	71.42	71.92	70.92	70.59	69.61	1.57	0.909	0.028	0.999
	CU	71.32	71.90	71.33	70.84	70.42				
	TL	70.51	70.75	70.47	70.61	69.27				
	TS	70.70	70.98	70.73	70.30	69.94				
MUFA	CS	24.69	24.28	24.93	25.12	26.21	1.39	0.945	0.196	0.978
	CU	24.85	24.31	24.69	24.97	25.53				
	TL	25.64	25.36	25.55	25.14	25.61				
	TS	25.27	25.13	25.36	25.55	25.50				
PUFA	CS	3.89	3.80	4.14	4.30	4.18	0.27	0.886	< 0.001	0.899
	CU	3.84	3.79	3.98	4.19	4.06				
	TL	3.85	3.89	3.97	4.25	4.67				
	TS	4.03	3.89	3.91	4.15	4.56				
SMCFA	CS	27.79	28.24	26.64	27.37	27.00	0.85	0.837	0.340	0.404
	CU	27.78	28.14	27.08	27.31	27.35				
	TL	28.08	28.53	27.41	27.06	26.87				
	TS	28.11	28.73	26.90	27.19	28.10				
LCFA	CS	34.23	34.43	37.99	36.17	36.40	0.85	0.863	< 0.001	0.842
	CU	34.51	34.34	36.72	36.08	36.33				
	TL	33.74	34.88	36.70	36.39	37.56				
	TS	33.99	34.91	36.70	36.19	38.67				

The control group of cows (CTL) received intramammary saline infusions in quarters of one udder half (CS), while the other half remained untreated (CU). The treatment group (LPS) received intramammary infusions of lipopolysaccharide in saline per quarter in one udder half (TL) and saline alone in quarters of the other half (TS). TRT = Treatment. Blood and foremilk samples were collected at −1 (0), 3, 6, 12, and 24 h relative to the intramammary challenge. Asterisks denote significance between treatments at specific time points (^**^P < 0.01, ^***^P < 0.001)

### Δ9 Desaturation index in plasma TAG

**Fig. 3:**
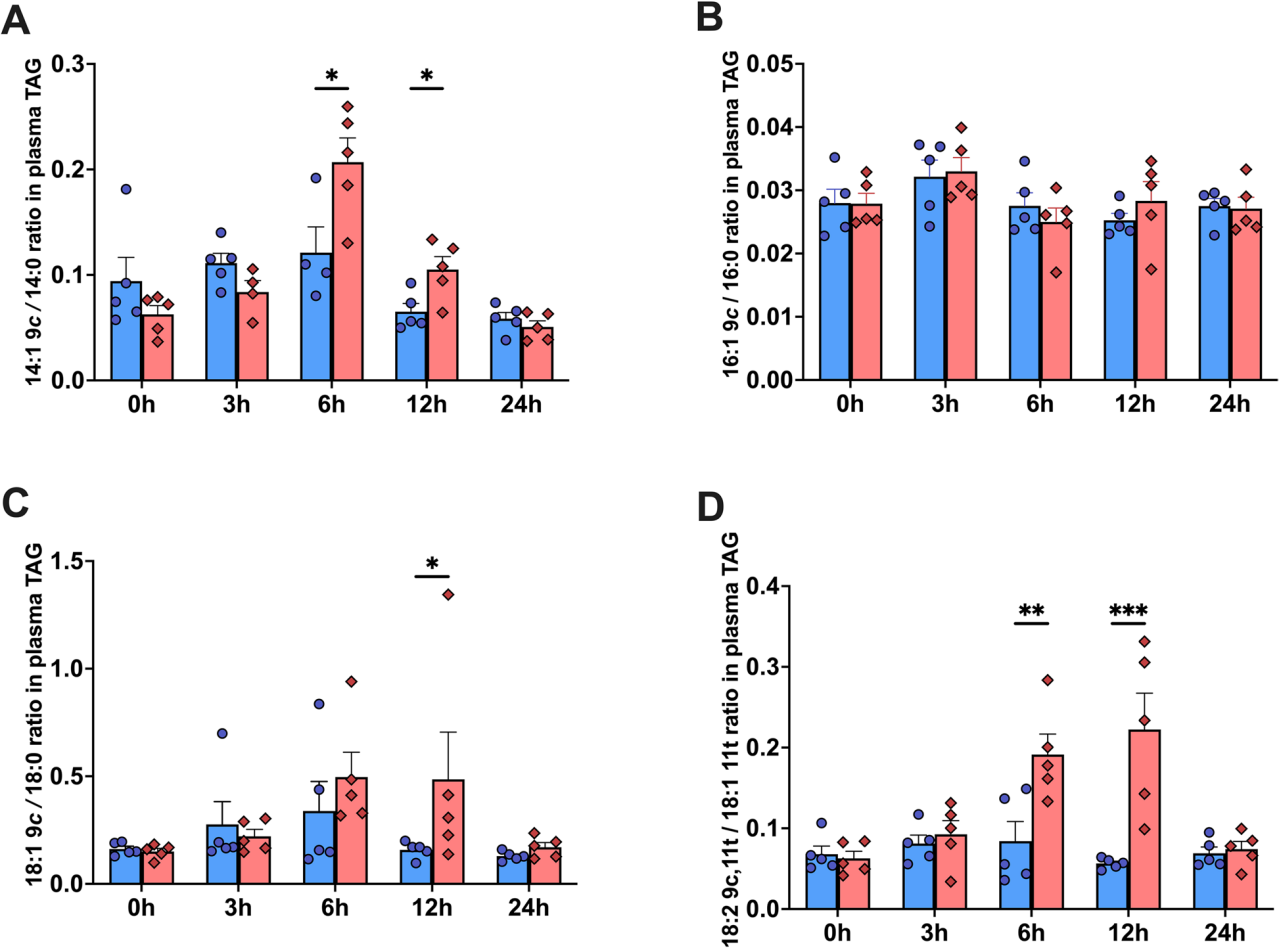
Δ9 Desaturation indices in plasma triacylglycerols (TAG) of dairy cows following an intramammary infusion of either saline (blue, *n* = 5) or lipopolysaccharide (red, *n* = 5). **A** 14:1 9*c*/14:0. **B** 16:1 9*c*/16:0. **C** 18:1 9*c*/18:0. **D** 18:2 9*c*,11*t*/18:1 11*t*. Error bars represent mean ± SEM. ^*^P < 0.05, ^**^P < 0.01, ^***^P < 0.001

Δ9 Desaturase activity was estimated using the ratios of 14:1 9*c*/14:0, 16:1 9*c*/16:0, 18:1 9*c*/18:0, and 18:2 9*c*,11*t*/18:1 11*t* in the plasma TAG fraction. Compared to the control group, LPS-treated cows exhibited increases in Δ9 desaturation indices: 14:1 9*c*/14:0 ratio increased 1.71-fold at 6 h (*P* = 0.039) and 1.62-fold at 12 h (*P* = 0.029), the 18:1 *9c*/18:0 ratio increased 3.08-fold at 12 h (*P* = 0.021), and the 18:2 9*c,*11*t*/18:1 11*t* ratio increased 2.27-fold at 6 h (*P* = 0.002) and 3.89-fold at 12 h (*P* < 0.001) (Fig. 3).

### Fatty acid composition of plasma PL

There was a treatment effect on the proportion of LCFA (*P* = 0.023) in plasma PL (Table 1). Additionally, a time effect was observed for the proportions of all classes of FA in plasma PL (*P* < 0.02), whereas a time × treatment interaction was detected for MUFA only (*P* = 0.044).

### Fatty acid composition of plasma CE

No treatment effect was observed on the proportions of any FA class in plasma CE (Table 1). However, a time effect was observed for SFA (*P* < 0.001), MUFA (*P* = 0.007), SMCFA (*P* = 0.01), and LCFA (*P* < 0.001). Additionally, a time × treatment interaction was detected for SFA (*P* = 0.002), SMCFA (*P* = 0.013), and LCFA (*P* < 0.003).

### FA profile of blood lipid fractions

**Fig. 4:**
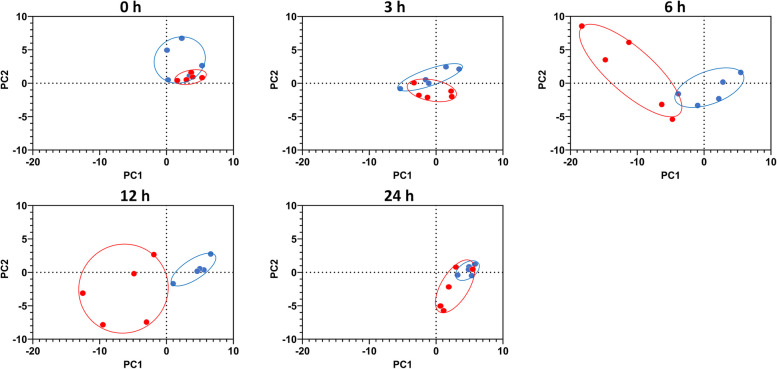
Principal component analysis of changes in fatty acid composition (g/100 g of fatty acids identified) in plasma triacylglycerols in dairy cows following an intramammary infusion of either saline (blue, *n* = 5) or lipopolysaccharide (red, *n* = 5) at the indicated time points. Proximity of points reflects similarity in fatty acid composition, and ellipses represent 95% confidence interval for each group. PC1 and PC2 accounted for 53.32% of the total variation in the data set

**Fig. 5:**
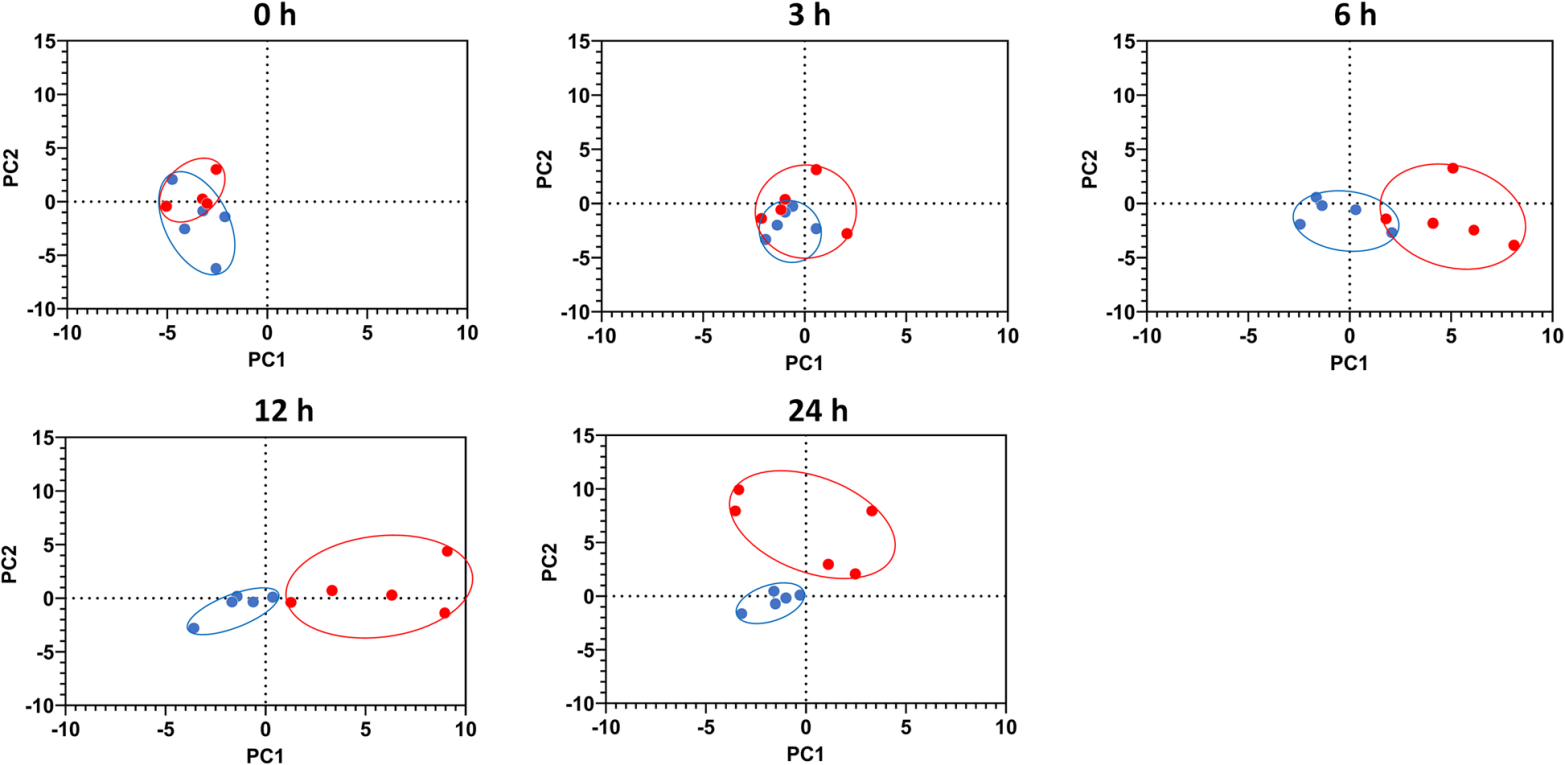
Principal component analysis of changes in fatty acid composition (g/100 g of fatty acids identified) in plasma phospholipids in dairy cows treated with an intramammary infusion of either saline (blue, *n* = 5) or lipopolysaccharide (red, *n* = 5) at 0, 3, 6, 12, and 24 h. Proximity of points reflects similarity in fatty acid composition, and ellipses represent 95% confidence location for each group. PC1 and PC2 explained 26.91% of the total variation in the data

Principal component analysis was performed to differentiate treatment groups based on the FA composition of plasma lipid fractions (TAG, PL, and CE). Due to overall low concentrations of FFA in the plasma of dairy cattle, FFA composition could not be properly assessed against the background noise for most samples. Principal component analysis was conducted at individual time points (0, 3, 6, 12, and 24 h) to visualize the differences between the treatment groups. The PCA plots were generated using the first two principal components. For plasma TAG, these components explained 53.32% of the total variation and revealed distinct clustering of samples by treatment at 6 and 12 h (Fig. 4). For plasma PL, the first two principal components accounted for 26.91% of variation, showing treatment-based clustering at 6, 12, and 24 h (Fig. 5). In contrast, FA composition in plasma CE did not exhibit clustering by treatment at any time point (data not shown).

### Plasma PGE_2_ concentration

**Fig. 6:**
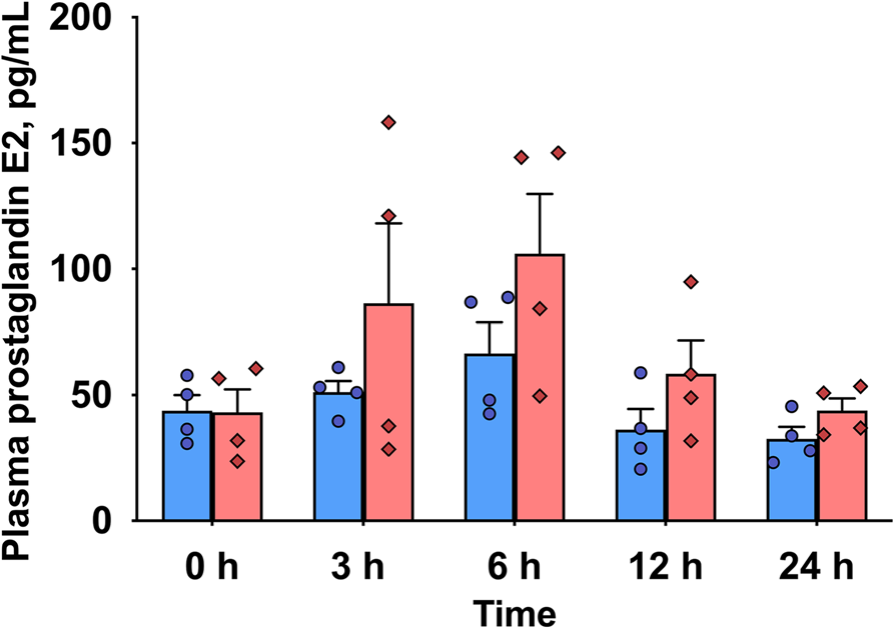
Plasma prostaglandin E_2_ concentrations in dairy cows following an intramammary infusion of either saline (blue, *n* = 5) or lipopolysaccharide (red, *n* = 5) at 0, 3, 6, 12, and 24 h. There were significant treatment (*P* = 0.027) and time (*P* = 0.021) effects, but no time × treatment interaction. Error bars represent means ± SEM

Plasma PGE_2_ concentration showed a treatment effect (*P* = 0.027) and time effect (*P* = 0.021; Fig. 6). However, no time × treatment interaction was observed.

### Fatty acid composition of milk

No treatment effect was observed for any FA class or individual FA in foremilk. However, there was a time effect for the proportions of SFA (*P* = 0.028), PUFA (*P* < 0.001), and LCFA (*P* < 0.001) (Table 1).

**Fig. 7:**
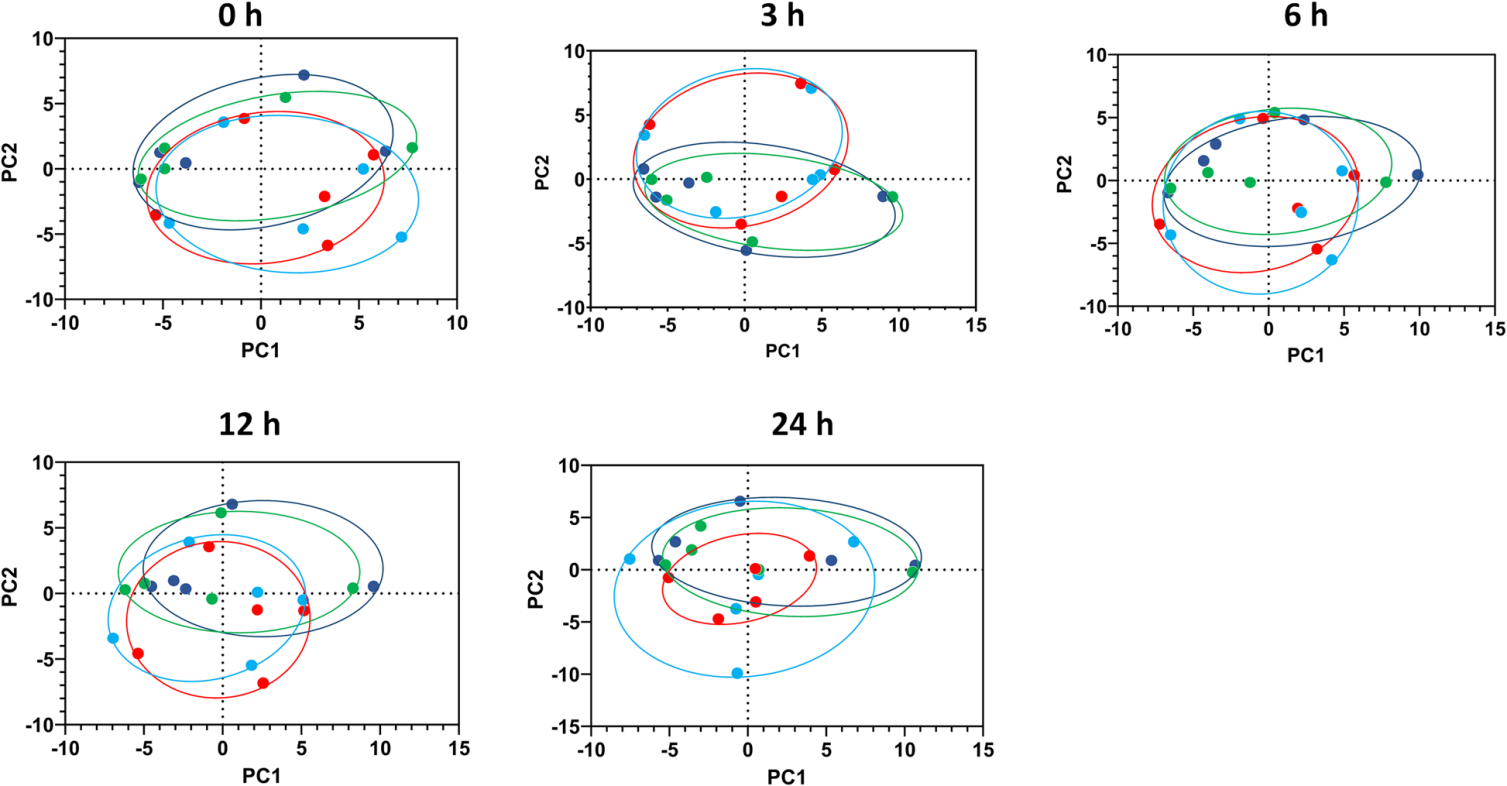
Principal component analysis of changes in fatty acid composition (g/100 g of fatty acids identified) in foremilk of dairy cows with or without an intramammary lipopolysaccharide (LPS) treatment for 0 to 24 h. The LPS-treated group received intramammary infusions of LPS in quarters of one udder half (red, *n* = 5) or saline alone in quarters of the other half (light blue, *n* = 5); the control group received intramammary infusions of saline in quarters of one udder half (dark blue, *n* = 5) and no treatment in the other half (green, *n* = 5). Foremilk samples were collected at the indicated time points following LPS or saline infusion. Proximity of points reflects similarity in fatty acid composition, and ellipses represent 95% confidence location for each group

Fatty acid composition in milk did not cluster by treatment group or by quarter in PCA, regardless of the first seven principal components, which collectively accounted for 82.05% of sample variation (Fig. 7). The first two components (PC1 and PC2) explained 45.05% of the total variance in the data set.

### Correlations between plasma PGE_2_ and FA in plasma TAG

Correlation analysis of plasma PGE_2_ with FA classes and individual FA in the plasma TAG fraction showed moderate positive correlations between plasma PGE_2_ concentrations and MUFA, PUFA, or < 16 C FA in plasma TAG (*r* = 0.33–0.52, *P* < 0.01). Three of the Δ9 desaturation indices (14:1 *c*9/14:0, 18:1 9*c*/18:0, and 18:2 9*c*,11*t*/18:1 11*t*) were moderately correlated with plasma PGE_2_ (*r* = 0.43–0.52, *P* < 0.001) (Table 2). Conversely, plasma PGE_2_ showed negative correlations with plasma TAG LCFA (*r* = −0.41, *P* < 0.01) and plasma TAG SFA (*r* = −0.51, *P* < 0.001) (Table 2).

**Table 2:** Pearson’s correlation coefficients between plasma prostaglandin E_2_ (PGE_2_) concentration and the fatty acid (FA) composition and Δ9 desaturation index (DI) in plasma triacylglycerols (TAG)

	Correlation coefficient with plasma PGE_2_	*P*-value
Plasma TAG Δ9 DI 14:1/14:0	0.45	< 0.001
Plasma TAG Δ9 DI 16:1/16:0	−0.05	0.690
Plasma TAG Δ9 DI 18:1/18:0	0.52	< 0.001
Plasma TAG Δ9 DI 18:2/18:1	0.43	< 0.001
Plasma TAG SFA	−0.51	< 0.001
Plasma TAG MUFA	0.52	< 0.001
Plasma TAG PUFA	0.33	0.009
Plasma TAG C < 16	0.33	0.008
Plasma TAG C > 16	−0.41	0.001

*SFA* Saturated FA, *MUFA* Monounsaturated FA, *PUFA* Polyunsaturated FA

## Discussion

Mastitis is well known to directly impair local mammary tissue’s ability to synthesize and secrete milk components, as well as to affect other distal tissues and organ systems in clinical cases [5, 31, 32]. In our previous companion study, we observed that intramammary LPS infusion led to 1.5- to 2-fold reduction in foremilk fat percentage at 3 and 6 h post-infusion. This effect was observed in both LPS-treated and untreated quarters of the treatment cows, suggesting systemic consequences and involvement [5]. However, the effects of mastitis on the FA composition of blood lipid fractions and milk are less known. In this study, we investigated changes in the FA composition of blood lipid fractions between cows following intramammary LPS or saline infusion, assessed changes in milk FA composition in individual udder quarters using a unilateral design, and looked for possible relationships between the changes in FA composition of blood lipid fractions and the changes in plasma PGE_2_ concentrations in response to LPS treatment.

### Impact on the concentrations of plasma lipid fractions

Despite a strong time-dependent effect on concentrations of both plasma TAG and FFA, no treatment effects or time × treatment interactions were observed in these two variables in the present study. The parallel increase in TAG and FFA concentrations in both groups during the first 24 h of the treatment suggests that these variations were unrelated to LPS and could be attributed to the experimental procedures, circadian rhythms, and the timing of animal feeding. Continuous intravenous LPS infusion in cattle initially increased circulating FFA (d 1–3), but decreased FFA from d 4 to 7 [33]. However, intravenous LPS administration in the study by Horst et al*.* [33] was markedly different from our model, where a single intramammary infusion likely resulted in minimal LPS leakage into circulation. Mastitis is known to increase mammary blood flow during the first 12 h [34], suggesting that the LPS-induced reduction in foremilk fat content observed in our previous study [5] may not be due to a decrease in circulating TAG or FFA availability.

### Fatty acid composition of plasma lipid fractions

We next determined the changes in the FA composition of each plasma lipid fraction. Principal component analysis revealed that the FA composition of plasma TAG clustered by group at 6 h and 12 h and this effect was absent by 24 h. In addition, the plasma PL FA composition also clustered by treatment at 6, 12, and 24 h, but only LCFA proportions were affected. This numerical decrease in LCFA (< 1.5%) in PL was minimal. Furthermore, since PC1 and PC2 accounted for only 27% of the total variation, specific FA shifts within the PL fraction could not be clearly identified. Plasma PL are a complex mixture of various lipid classes, and the observed clustering may indicate subtle, undetected changes in specific subclasses. Future studies using targeted lipidomics could provide a clearer understanding of these shifts.

The major specific FA changes in plasma TAG at 6 and 12 h of LPS treatment included: i) the rises in Δ9 desaturation indices for C14 and C18, ii) the increase in SMCFA and decrease in LCFA (C18 and C20), and iii) increases in n-3 and n-6 FA. The effects on Δ9 desaturation indices indicate a potential change in stearoyl-CoA desaturase 1 (SCD1) activity in hepatocytes and adipocytes. The Δ9 desaturation index serves as a proxy for SCD1 activity, a key enzyme in FA and TAG metabolism [35, 36]. SCD1 catalyzes the introduction of a double bond at the 9^th^ position in medium- and LCFA, converting SFA or MUFA to MUFA or PUFA. However, a puzzling discrepancy was that the desaturation index based on C16 showed no difference between treatments, whereas the other three indices showed a significant treatment effect. This could be because palmitic acid (16:0) is the primary FA synthesized de novo, and a constant resupply of 16:0 might compensate, at least in part, for the conversion to 16:1. Another possible explanation is the presence of various isoforms of the SCD enzyme [37] which may have preferences on the length of FA and be regulated differently during inflammation. In addition, the increase in SMCFA and decrease in LCFA may suggest an increase in the oxidation of LCFA to release more SMCFA in hepatocytes and/or adipocytes. Furthermore, the increases in n-3 and n-6 FA may imply possible changes in the activities of other desaturases (such as Δ4 and Δ5 desaturases) involved in the synthesis in these FA [38].

The changes in FA of plasma TAG were likely mediated by LPS-induced systemic changes in cytokines and other inflammatory mediators, such as oxylipins including PGE, acting on hepatocytes and adipocytes. It is well known that lipid metabolism is regulated during the host response to inflammation, mediated by cytokines [39, 40]. Our previous companion study showed that intramammary LPS infusion quickly increased plasma IL-6, IL-10, IP-10, MCP-1, and MIP-1β [41], among which IL-6 and MCP-1 are known regulators of lipid metabolism [42, 43]. It has been shown that IL-6, which is both pro- and anti-inflammatory, inhibits the activation of the peroxisome proliferator-activated receptor gamma (PPAR-γ), a master regulator of adipogenic differentiation and cell metabolism [44, 45]. The chemokine MCP-1 increases liver lipid accumulation by up-regulation of sterol regulatory element-binding protein 1 (SREBP-1c), a key regulator of lipogenesis [46]. However, the effects of inflammatory mediators on lipid metabolism and their underlying mechanisms in mastitis have not been studied to our knowledge. From our observations, we hypothesize that the activity of SCD1 and other desaturases involved in n-3 and n-6 FA synthesis is regulated by inflammatory mediators, one of which is potentially PGE_2_ whose blood concentrations were positively correlated with three Δ9 desaturation indices in the present study. Due to the shared synthesis pathways of many eicosanoids, it is likely that prostaglandin D_2_, prostaglandin F_2α_ (PGF_2α_), prostacyclin (PGI_2_), and thromboxane A_2_ play a role in the pathophysiology of mastitis [47]. Previous studies have established that PGF_2α_ and TXA_2_, both pro-inflammatory eicosanoids, are positively correlated with SSC in milk during mastitis [48]. While milk PGF_2α_ concentration negatively correlates with milk yield [48], several other eicosanoids have been shown to be elevated in both milk and plasma during mastitis [23]. However, their specific roles in the pathophysiology of mastitis and the underlying mechanism remain unclear.

A possible mechanism behind the marked increase of the proportion of PUFA in TAG is the mobilization of phospholipids. As shown in the present study, the PL fraction was much richer in PUFA than the TAG fraction; PUFA constituting between 36% and 40% of total PL FA. In humans, IL-6 is a potent activator of phospholipase A2 expression in the liver [49]. One role of phospholipase A2 is to hydrolyze PL to liberate arachidonic acid (20:4 5*c*,8*c*,11*c*,14*c*) and other PUFA [50]. We hypothesize that the liver responds to the circulating inflammatory cytokines with increased phospholipase activity. This in turn increases the pool of PUFA available, which are incorporated into TAG as the liver is synthesizing them for packaging into very-low-density lipoprotein (VLDL).

It is particularly relevant that the PUFA proportions in plasma TAG increased drastically at 6 and 12 h post-LPS infusion because several PUFA, including linoleic (18:2 9*c*,12*c*), arachidonic (20:4 5*c*,8*c*,11*c*,14*c*), eicosapentaenoic (20:5 5*c*,8*c*,11*c*,14*c*,17*c*), and docosahexaenoic (22:6 4*c*,7*c*,10*c*,13*c*,16*c*,19*c*) acids, are known to regulate the synthesis of oxylipins [23]. Oxylipins, also referred to as eicosanoids in animals [51], are oxygenated PUFA metabolites that serve as potent inflammatory mediators and play diverse biological roles [24]. The increase in various n-6 FA, such as 18:2, 18:3, and 20:3, is particularly noteworthy, as these FA are precursors of arachidonic acid, which in turn serves as the precursor of pro-inflammatory prostaglandins, including PGE_2_ [23, 52–54]. In addition, various n-3 FA, specifically 20:3 and 20:5*,* were also elevated. Oxylipins derived from 20:5 n-3 and 22:6 n-3 include bioactive mediators such as resolvins, lipoxins, and protectins, which are generally considered more anti-inflammatory than oxylipins derived from linoleic and arachidonic acid. They tend to counteract the effects of n-6-derived oxylipins and promote inflammation resolution [55, 56]. However, as previously demonstrated, oxylipins cannot be strictly categorized as either pro- or anti-inflammatory [57], as their effects depend on specific receptors they activate, which can have opposing effects [25].

We measured plasma PGE_2_, the most abundant prostaglandin, as a potential mediator of inflammation and blood and milk lipid composition [58]. Prostaglandin E_2_ is a potent vasodilator [12]. It also plays an important role in immune regulation, influencing the activation, maturation, migration, and cytokine secretion of immune cells [58, 59]. Our results confirmed that LPS treatment increased plasma PGE_2_ concentrations in cows. These findings align with previous studies by Ryman et al. [60] and Filor et al*.* [61] which demonstrated that n-6-derived eicosanoids increase during mastitis, and that mastitis leads to elevated PGE_2_ concentrations in mammary tissue. Collectively, these results support a role of PGE_2_ as an important inflammatory mediator in the pathophysiology of bovine mastitis.

Prostaglandins have been implicated in lipid metabolism, although much of this research is outdated [62–64]. Eicosanoids are also known to act in conjunction with cytokines and are particularly involved in signaling to the liver [65–67]. Oxidized lipids induce the production of MCP-1 [68]. Similar to IL-6, PGE_2_ inhibits the activity of PPAR-γ in adipocytes and lipogenesis in the liver [69, 70]. Concentration of eicosanoids can even act as biomarker of liver fibrosis and function [71, 72]. In humans, blood FA profiles correlate with hepatic function [73], and a similar mechanism is likely at play in cattle, given the liver’s central role in systemic lipid metabolism.

Another possibility to explain the changes of FA profile of blood TAG in LPS-treated cows is that the inflammatory mediators signal the liver cells to package different forms of lipoproteins. Evidence has shown that prostaglandins inhibit very low-density lipoprotein VLDL formation [62, 72]. This may explain some of the observed FA shifts in our study as lipoproteins have different lipid and FA compositions [74].

The reason that significant changes in FA profiles in blood were observed in the TAG fraction but not in CE and PL is likely because TAG are less abundant than either CE or PL in blood. In dairy cows, plasma TAG concentration is between 4% and 12% of the CE and PL fractions [75, 76]. Moreover, both CE and PL are already rich in PUFA. Taken together, these two facts might have masked any potential effect on the FA in CE and PL fractions.

### Milk fatty acids

It is generally accepted that milk FA with more than 16 carbons are derived from the bloodstream from hydrolysis of TAG in chylomicrons and in very-low density lipoproteins by lipoprotein lipase in the capillaries [77, 78], and milk FA less than 16 carbons are almost entirely synthesised de novo in the mammary gland [10, 79]. However, despite pronounced changes in the FA composition of plasma TAG, corresponding changes were not observed in milk FA composition in the present study. This suggests that the mammary gland actively regulates milk fat synthesis and secretion, mitigating fluctuations in plasma TAG FA composition. This aligns with previous studies that reported no changes in milk FA composition following mastitis [17, 80]. Several mechanisms could explain this phenomenon. First, suppressed milk fat synthesis resulted in little opportunity for fat to be influenced by altered plasma FA composition. Second, it is worth noting that the bovine mammary gland takes up most LCFA from the TAG in chylomicrons and VLDL [77, 78]. Chylomicrons originate directly from intestinal epithelial cells, making their composition highly sensitive to the diet. As the diet did not change, the changes seen in total plasma TAG might not be uniformly distributed across all lipoproteins. Further studies could investigate changes in FA composition of specific lipoprotein fractions to see if changes in the FA composition of chylomicrons would be reflected in the milk. Third, another possible mechanism would be that the specific expression pattern of fatty acid transport proteins, a family of six proteins essential to the uptake of LCFA, tightly regulates the identity of the LCFA that enter the cells. Finally, it is also possible foremilk, rather than total milk, did not reflect acute changes in FA composition of milk fat. While it has been previously reported that the first 100 mL of milk is representative of total milk in terms of FA composition [80], alveolar milk also has a much higher fat content than cisternal milk [81].

Lastly, we recognize a limitation in our study. Due to the very low concentration of many of the FA in the plasma FFA fraction, we were unable to reliably measure the FA composition of the plasma FFA fraction despite repeated attempts. We acknowledge that FFA can be another important source of milk LCFA and are an indicator of systemic lipid metabolism.

## Conclusions

Intramammary LPS infusion induced pronounced alterations to plasma lipid but had no impact on milk lipid composition within the first 24 h. We observed a shift in plasma TAG FA composition including an increased proportion of linoleic acid and other PUFA, along with an increased Δ9 desaturation indices based on C14 and C18. Additionally, plasma PGE_2_ concentrations increased following intramammary LPS infusion and were positively associated with three of the four Δ9 desaturation indices evaluated. These findings highlight the complex interplay between whole body and mammary gland lipid metabolism during mastitis and suggest a potential role of PGE_2_ and SCD enzymes in the pathophysiology of mastitis.

## Fatty acid nomenclature

Palmitic acid: 16:0

Oleic acid: 18:1 9*c*

Linoleic acid: 18:2 9*c*,12*c*

Arachidonic acid: 20:4 5*c*,8*c*,11*c*,14*c*

Eicosapentaenoic acid: 20:5 5*c*,8*c*,11*c*,14*c*,17*c*

Docosahexaenoic acid: 22:6 4*c*,7*c*,10*c*,13*c*,16*c*,19*c*

## Data Availability

The datasets used and/or analysed during the current study are available from the corresponding author on request.

## Abbreviations

CE: Cholesterol esters
DI: Desaturation index
FA: Fatty acids
FFA: Free fatty acids
LCFA: Long-chain fatty acids
LPS: Lipopolysaccharide
MUFA: Monounsaturated fatty acids
NEFA: Non-esterified fatty acids
PCA: Principal component analysis
PGE_2_: Prostaglandin E_2_
PL: Phospholipids
PPAR-γ: Proliferator-activated receptor gamma
PUFA: Polyunsaturated fatty acids
SCD1: Stearoyl-CoA desaturase 1
SFA: Saturated fatty acids
SMCFA: Short- to medium-chain fatty acids
SREBP-1c: Sterol regulatory element-binding protein 1c
TAG: Triacylglycerides
